# Being kind in unkind spaces: a qualitative examination of how medical educators and first year medical students perceive empathy training

**DOI:** 10.3389/fsoc.2023.1272357

**Published:** 2024-01-19

**Authors:** Sarah D. C. Harvey, Clare L. Stacey

**Affiliations:** Department of Sociology and Criminology, Kent State University, Kent, OH, United States

**Keywords:** clinical empathy, empathic capital, empathic dissonance, affective practice, emotion practice, medical students, medical educators

## Abstract

**Introduction:**

It has become *de rigueur* for healthcare systems to tout their ability to provide compassionate medical care that addresses the emotional as well as physical needs of patients. Not surprisingly, then, there is considerable pressure on medical schools to train their students to be empathic. Existing literature on empathy training in medicine tends to focus on how to build emotional intelligence in individual trainees, largely ignoring the sociocultural factors that contribute to or thwart empathy development in medical school. Additionally, research tends to examine student perspectives, with little attention given to medical educators and their viewpoints.

**Methods:**

In this paper, we adopt an “emotion practice” framework and utilize an inductive descriptive study design to qualitatively consider how first year medical students (*N* = 23) and their instructors (*N* = 9) perceive empathy training at a site we call Midtown Medical School.

**Results and discussion:**

We find that both groups have an understanding of empathic capital but differ in their beliefs about the utility and legitimacy of this capital. Both educators and students also recognize the limitations of standardized empathy curriculum but do not agree on the implications of such rote learning. Finally, students and instructors alike find the hidden curriculum of medical school to be antithetical to empathy development, concurring that it is difficult to cultivate empathy in spaces where biomedical coursework is prioritized over social–emotional learning. In short, both groups find it difficult to *be kind in an unkind place*.

## 1 Introduction

The development of clinical empathy (CE) in medical trainees is viewed as a necessary component of cultivating a compassionate healthcare workforce. Indeed, it has become *de rigueur* for healthcare systems to tout the degree to which providers and services are compassionate, caring, and focused on the emotional—as well as physical—well-being of patients. Not surprisingly, then, there is considerable pressure on medical schools to train their students to be empathic actors. These efforts are not simply cynical attempts at securing patient market share. There is ample evidence that CE can improve patient satisfaction and treatment compliance and can also reduce burnout for providers (Eisenberg and Fabes, 1990; Jeffrey, 2016; Tan et al., 2021).

Most medical schools engage in some form of empathy training, ranging from narrative medicine courses to explicit training using simulated patients and mock interviewing. These interventions appear to improve patient-centered communication skills and enhance empathy among trainees (Batt-Rawden et al., 2013; Underman and Hirshfield, 2016; Kataoka et al., 2019; Seeberger et al., 2020). Training tends to focus on enhancing the emotional intelligence of *individual* medical students to provide them with skills to enact empathy during clinical encounters. This conceptualization of CE, while necessary, overlooks the sociocultural dimensions at play in empathy training, such as the emotional labor involved in enacting empathy; the emotional capital students bring to the table when matriculating into medical school; and the hidden curriculum of medical school that values clinical and biomedical training over the relational or interactional (Smith and Kleinman, 1989; Hafferty, 1998; Underman, 2015; Vinson and Underman, 2020; Harvey et al., 2023).

Building on these sociocultural analyses of CE, the current paper considers how both medical students and their instructors perceive empathy training at a site we call Midtown Medical School (a pseudonym). Combining student and educator perspectives allows for an understanding of how perceptions of empathy training overlap and converge in these two populations, something understudied in the literature (see Vinson and Underman, 2020 for a notable exception). We find that students and faculty agree that empathy training is often scripted, rote, and at times disingenuous, but their respective interpretations of these realities differ. Faculty utilize a “fake it until you make it” framework for understanding CE training, whereas students believe they already possess adequate emotional capital to enact CE in “real” and meaningful ways. Students and educators do agree that the speed up of medical school and the hidden curriculum of training—namely, value placed on clinical and biomedical courses—undermines the development of CE. In short, both groups argue that it is very difficult to *be kind in an unkind space*.

## 2 Literature review

Clinical empathy, as defined by scholars of medical education, is understood as “the ability to understand the patient’s situation, perspective, and feelings, while also communicating that understanding to the patient and acting on that understanding in a helpful way” (Mercer and Reynolds, 2002, S9). Empathy prompts helping behavior (Shott, 1979) and is found to reduce burnout in providers and positively impact patient satisfaction and patient distress (Eisenberg and Fabes, 1990; Jeffrey, 2016; Tan et al., 2021). Medical educators tend to privilege cognitive over affective (felt) empathy, as it is believed cognitive empathy guards against provider loss of objectivity (Coulehan, 1995; Markakis et al., 2000; Mercer and Reynolds, 2002; Bloom, 2016; Hojat, 2016). It is worth noting that this rigid distinction between cognition and emotion is itself up for debate in the literature (Lief and Fox, 1963; Halpern, 2001; Underman and Hirshfield, 2016; Ajjawi et al., 2022; Cottingham, 2022).

Empathy training includes approaches such as narrative medicine, creative arts, verbal and nonverbal communication skills training, interpersonal skills training, and experiential learning (Batt-Rawden et al., 2013). Notably, in the area of verbal communication skills training, medical students participate in seminars about empathic interviewing and practice what they learn in simulated interactions with standardized patients (SPs) and peers (Laughey et al., 2020a, 2021; Brodahl et al., 2022; Tengiz et al., 2022; Underman et al., 2022). Generally, educators perceive standardized and simulated interventions as effective for improving patient-centered communication skills and enhancing empathy among health professions trainees (Batt-Rawden et al., 2013; Bearman et al., 2015; Engbers, 2020; Tengiz et al., 2022).

The medical education literature tends to view CE through an individualist lens, conceptualizing it as a form of emotional intelligence that medical students can learn to cultivate and enact through explicit training. While CE is of course individually mediated, sociologists who study empathy in medicine suggest there are also meso- and macro-level forces at play. Specifically, sociocultural arguments about CE cluster around three key claims: that CE is a form of emotional labor; that it is a resource (capital) in addition to being a felt and expressed emotion state; and that it is cultivated in the context of an environment where the informal curriculum downplays the relevance and importance of emotional socialization and training. We consider each of these sociocultural critiques below and argue that, taken together, they constitute an emotion practice approach (Cottingham, 2022) that is invaluable to understanding how medical students learn and practice empathy.

### 2.1 Clinical empathy as emotional labor

Clinical empathy can be considered a form of emotional labor, as providers and trainees are encouraged to mold themselves into empathic actors who can and should regulate the nature of provider-patient interactions (Hochschild, 1983; Larson and Yao, 2005; Vinson and Underman, 2020; Brodahl et al., 2022). In this sense, empathy becomes a type of “affective practice” (Underman, 2015, p. 180) wherein students learn how to “reshape the body’s capacity” to manage emotion while interacting with patients. Not all scholars agree on the implications of CE as emotional labor. Larson and Yao (2005) argue that CE as emotional labor ultimately enhances clinical practice since *acting* empathic will ultimately lead to *genuine* displays of empathy, whereas other scholars caution against the consequences of organizational dictates on feeling that ultimately benefit the institution of medicine over and above providers and patients (Vinson and Underman, 2020). Wear and Varley (2008) express concerns about the “simulation game” in particular and how it encourages performative, inauthentic empathy among medical students. Similarly, a number of studies show that students themselves find fault with empathy training, specifically the use of blanket empathetic statements, simulated or “fake” interactions, and the overall assessment and quantification of empathy, all of which are seen to diminish the value of students’ “natural” empathic tendencies and “reduce human connection to programmable modalities” (Hafferty et al., 2015; Perrella, 2016, 2; Jeffrey, 2019; Laughey et al., 2020a,b, 2021; Brodahl et al., 2022; Underman et al., 2022).

### 2.2 Empathic capital, socialization, and habitus

Medical training and socialization can also be examined through Bourdieu’s concepts of field, habitus, and capital (Bourdieu, 1984). Such a formulation posits that within the field (i.e., institution) of medicine, students and clinicians possess and reproduce a set of norms, dispositions, and behaviors (habitus) related to the display of emotion in clinical encounters. This habitus is informed by skills and resources (capital) that are accrued in the context of primary (e.g., the family, K-12 education) and secondary (e.g., professional training) socialization. Emotional capital is understood as “knowledge, contacts, and relations, as well as access to emotionally valued skills and assets” used to “generate emotional perceptions, reactions, expressions, and emotion management strategies across various situations” (Cahill, 1999, 112). The advantage of a Bourdieuian approach to empathy training is that it places medical student learning within a larger organizational and institutional context, to better understanding how student orientations to felt and displayed empathy are conditioned by past and current socialization.

A student’s “emotional habitus” (Gould, 2009)—habits, skills, and dispositions—is profoundly influenced by capital accrued via primary and secondary socialization (Cahill, 1999; Erickson and Stacey, 2013; Underman, 2015; Jenkins et al., 2018; Cottingham and Erickson, 2019; Cottingham, 2022; Harvey et al., 2023). Harvey et al. (2023) find that pre-medical students matriculate to medical school with extensive knowledge and skills related to understanding the emotion states of others, what the authors dub “empathic capital.” Students also accrue empathic capital as they move through pre-clinical coursework that instructs them on relational skills such as patient interviewing and delivering bad news. The trouble is that student emotional capital is rarely acknowledged in the context of medical training. As Wacquant (2014) cautions, if the habitus developed in primary socialization by trainees is vastly different from the parameters of the habitus required of their experiences in secondary socialization, “the more difficult the traineeship… and the less integrated the resulting dispositional formation is likely to be” (Wacquant, 2014, 8; Underman, 2015).

Indeed, current literature documents the difficulties students face as they bring their own empathic capital to curricular interventions such as simulated patient training (Jeffrey, 2019; Laughey et al., 2020a,b; Brodahl et al., 2022). Brodahl et al. (2022) warn of a “moral dilemma” that potentially results from asking medical students to perform empathy or minimize their operating understandings of empathy. Additionally, Laughey et al. (2021, p. 1942) find that medical trainees experience instances of empathic dissonance in the context of empathy training: “the mental discomfort experienced by the act of making expressions of empathy that are not sincerely felt.” How students then resolve this dissonance—and how their instructors acknowledge or support them in these efforts—is a crucial empirical question.

### 2.3 Empathy and the hidden curriculum

In addition to the problem of empathic dissonance, medical school is an environment that simultaneously promotes the importance of relational skills training while also indirectly communicating that performance in biomedical courses is of paramount importance. These conflicting messages about the value of empathy are generally not made explicit but are rather conferred in the interstices of medical training, via informal conversations with superiors and peers and through cultural spaces like internet discussion boards. Medical sociologists refer to this as the informal or hidden curriculum (Hafferty, 1998). Jeffrey (2019) finds medical school curriculum to be a “barrier to empathy” such that even when medical schools outwardly prioritize creating “caring competent doctors,” students are well aware that their focus should be on the accumulation of biomedical knowledge above all else (Jeffrey, 2019, p. 168). A student in the study of Jeffrey (2019, p. 168) notes that once you have successfully dealt with the “biological stuff,” then perhaps you can consider things like “empathizing with patients.”

Further, Hafferty et al. (2015) observe that squeezing “on doctoring” courses into already arduous student schedules sends a message to trainees that learning the fundamentals of doctor-patient interaction is not actually valued. Students also grapple with variability in course content, materials, and the educators themselves, who may contradict course learning objectives or act as unempathetic role models (Hafferty et al., 2015; Underman and Hirshfield, 2016). Students, thus, are faced with the task of ascertaining on their own what is valuable and what is not valuable in medical training. But how do educators feel about the informal curriculum, especially when it comes to CE training? And how do they train students in empathy, given the priorities placed on biomedical training? These questions are relatively unexplored in the existing literature on medical education, something the current paper attempts to address.

### 2.4 Clinical empathy training as emotion practice

As the above literatures make clear, training students to engage in CE with patients amounts to more than imparting a set of discrete communication skills to individual students in the hopes that it will improve doctor-patient interaction. Significant contextual and sociocultural factors are at play when students are asked to learn and enact CE. We suggest that integrating these various sociocultural views into an understanding of CE constitute what sociologist Cottingham (2022) calls an emotion practice approach. Such a formulation highlights three things about CE: First, emotion practice (EP) recognizes that empathy is a resource or form of capital that can be called on to foster connection to others but can also deplete the emotional reserves of social actors and lead to empathic dissonance, similar to other forms of emotional labor. Second, emotion practice positions CE within an understanding of emotional habitus (Gould, 2009, p. 32) wherein we are able to examine how student “dispositions” about empathy are formed before students get to medical school and also while they navigate pre-clinical and clinical experiences. Finally, EP calls into question the dualisms inherent in the hidden curriculum of medical school that privilege reason over emotion and indirectly communicate to students that biomedical training is separate from (and more valuable than) relational skills. This last point is particularly germane, because—as Cottingham (2022, p. 33) points out—emotion and reason are only analytically distinct. In practice, emotions are always intertwined with so-called rational cognitive process (Feldman Barrett, 2017).

This paper utilizes an emotion practice approach to make sense of the points of overlap and divergence in educator and student perceptions of CE training. We find that both groups have a language and understanding of empathic capital, but they differ in their beliefs about the utility and legitimacy of this capital. Moreover, both educators and students recognize the limitations of standardized empathy curriculum but do not agree on the implications of a rote approach to emotional socialization. Finally, students and instructors alike view the environment of medical school to be antithetical to empathy development, agreeing that it is difficult to cultivate kindness in an unkind place.

## 3 Methods

Data for this paper are drawn from a 5-year longitudinal study that follows a cohort of United States pre-medical students (*N* = 23) from their last year of pre-medical training through their final year of medical school (2019–2023). The goal of the study is to ascertain how students conceptualize and practice empathy over time, i.e., their “empathy careers” (Ruiz-Junco, 2017, p. 426). We utilize an inductive, descriptive study design (Sandelowski, 2000), rooted in the constructivist paradigm (Charmaz, 2014; Creswell and Poth, 2016) that traces students’ perceptions and experiences via semi-structured interviews. The authors also interviewed medical educators (*N* = 9) in year 4 of the study to triangulate student accounts and to better understand the intentions behind the curriculum at Midtown Medical School. We received IRB approval from our home institution in February of 2019 for medical student interviews and in October of 2021 for interviews with MEs.

Respondents for this study were recruited in their second year of pre-medical education at Midwest University. The sample (*n* = 23) is drawn from a small population (*N* = 46) of Early Assurance (EA) students at Midwest, who completed their Bachelor of Science degrees and then matriculated into Midtown Medical School as part of an agreement between the two institutions. We intentionally sought a convenience sample of EA students for our longitudinal study because EA students are (1) more likely to matriculate to medical school relative to their non-EA peers and (2) are guaranteed acceptance into Midtown for medical school as a benefit of the EA program. The EA program provided a sample of students we could follow over-time and who all attended the same medical school. For the initial phase of the study, a recruitment email was distributed to the pre-medical program advisor at Midwest, who then distributed the email to students. Twenty-five students responded with interest in 2019, and we scheduled interviews with those students. Students who participated in this first phase of the study in 2019 were recruited by email for follow-up interviews in April of 2020. Twenty-three M1s responded with interest in year 2 of the study and interviews were scheduled with those students. With respect to medical educators (MEs), we sought a convenience sample from core faculty at Midtown involved in teaching doctor-patient communication skills to students (*N* = 23). MEs were contacted via email in April of 2022, and invited to participate in the study. Nine MEs responded with interest, and interviews were scheduled with those MEs.

Two of the 5 years of this study took place during the COVID pandemic. As such, the researchers utilized both in-person and Zoom technology to conduct interviews, depending on the preferences of the respondent and the public health recommendations of the day. At the time of each interview, participants were guided through an informed consent document detailing the nature of participation, the objectives of the study, as well as any potential risks and benefits. Written consent was obtained from all participants for their participation and for the use of transcribed interview data in research, publication, and presentation at professional meetings.

Interviews with both students and educators followed a semi-structured format, guided by interview schedules. For M1s, questions pertained to (1) their experiences with the biomedical curriculum, (2) the primary stressors they face as M1s, and (3) their perceptions of emotions and empathy, both personally and in the context of curriculum. For MEs, questions pertained to (1) their motivations for becoming MEs, (2) the rewards and challenges of working with medical students, and (3) their perceptions of emotions and empathy, both personally and in the context of curriculum. Interviews lasted 70 min on average, and participants received a $10 gift card in exchange for their time.

Interviews were audio recorded and then transcribed verbatim using *Otter.ai*. Transcriptions were subsequently reviewed for errors, de-identified, and pseudonyms were assigned to all locations, persons, and to all M1 and ME participants. Transcriptions were then uploaded to *Dedoose*, a qualitative data analysis software program. Data analysis was completed in multiple stages by the authors, and in each stage, a thematic analytic approach was applied to the data (Braun and Clarke, 2006). As interview data were collected, the authors engaged in initial coding to generate an exhaustive list of themes from the data (Charmaz, 2014; Saldaña, 2016). We created codes that were pertinent to each line or unit of data, primarily applying descriptive and *in vivo* codes (Charmaz, 2000; Chenail, 2012; Saldaña, 2016). Once we generated an exhaustive list of codes from all interview transcripts, the authors began the focused coding stage. In this stage, we merged codes that were conceptually the same, and grouped the remaining codes into broader thematic categories, relying on analytic discussions (between authors) and memoing to help refine the categories (Charmaz, 2014; Saldaña, 2016). We then compared the themes from our analysis to the existing sociological, psychological, and medical literatures on empathy, emotional socialization, and health professions education (Morse, 2020) to understand how our results contribute to an ongoing discussion about the nature and efficacy of empathy training. It is worth noting that since this is a longitudinal study with a discrete sample of students followed over 5 years, we knew from the outset that the standards of theoretical saturation might be difficult to meet (i.e., collecting data until no new themes appear). We were limited to the interview data generated by the 23 students (and nine educators) willing to be followed for such a long period of time. However, we did in fact reach theoretical saturation with the student sample, as no new themes emerged in the last third of interviews conducted. We did not reach theoretical saturation with the medical educators so focus in this paper on the themes we did identify across all nine respondents.

Of the 23 M1s discussed in this paper, 14 are women and nine men. The average age of M1 participants is 22 years old. Nine of our M1 respondents identify as white, 10 as Asian, one as African American, and three as biracial: white/Asian. Among the nine MEs, five identify as women and four as men. The average age of ME participants is 50. All MEs identify as white.

True to the constructivist tradition, we recognize the need to reflect on how our social locations and positionality shape the collection and analysis of data (Charmaz, 2014). The first author is a graduate student of sociology and the second an Associate Professor of Sociology at the same academic institution. Both share research interests in medical sociology, sociology of emotions, and sociology of health professions and health professions education. As such, we are in possession of substantial knowledge regarding the sociological examination of emotional socialization of healthcare professionals and trainees and tend to favor constructionist perspectives that emphasize the institutional and organizational contexts of felt and expressed emotion. As we analyzed data, we were careful to bracket our interpretations of emotional socialization, empathy, and empathy training from those of our participants (Lincoln and Guba, 1985). The second author teaches a medical humanities course to M1-M4 students at Midtown and also instructs pre-medical students in sociology courses at Midwest University. These responsibilities enabled the author to quickly develop rapport with research participants. The first author lives in medical student housing at Midtown Medical School, which allowed the author to establish and maintain ties with medical students *in situ*. In short, the authors are no strangers to the world of medical training, something that facilitated recruitment of research subjects and enriched data analysis. The respective roles of the two authors—doctoral student and tenure track faculty—helped ensure analytic integrity and trustworthiness (Lincoln and Guba, 1985) of data in the sense that each could provide a check on the others’ partial view of empathy training at Midtown. The first author provided an important sensitivity to the student experience and the second author an understanding of the constraints facing instructors. Similarly, each author could challenge what she believed to be a partiality in the others’ interpretation of data that stemmed from occupying the student or educator role.

## 4 Results

Drawing on data collected with first year medical students (M1s) and medical educators (MEs), we find that both groups have a language and understanding of empathic capital, but that they differ in their beliefs about the utility and legitimacy of this capital. Further, educators and students concur that there are limits to a standardized empathy curriculum but do not see eye to eye when it comes to the implications of a rote approach to emotional socialization. Finally, students and instructors alike view the environment of medical school to be antithetical to empathy development, agreeing that it is difficult to cultivate kindness in an unkind place.

### 4.1 Medical students’ perceptions of empathy curriculum

Consistent with the literature on medical students’ experiences with interview skills curriculum, 17 M1s in our study find fault with the empathetic statements they are instructed to use in the context of interview skills training and SP interactions. They refer to their experience delivering these statements as “fake,” “forced,” “robotic,” and “formulaic.” For example, Lailah recalls how MEs (and the curriculum) “make everything feel really robotic.” She goes on to say, “there’s jokes about it online… People are like, ‘here’s an M1 doing a standardized patient interview. If the patient is saying they are hav[ing] a heart attack; the M1 is just going to say ‘Oh, that must be frustrating’ instead of actually do[ing] something about it.’”

Echoing Lailah’s observations, Jabarr discusses how MEs “really drilled the idea of empathy” into them (he and his classmates). Recounting his experiences with empathy assessment he says, “I almost feel like they forced empathy, though. They’re like, you need to have three examples of empathy in every interview (with SPs).” From Jabarr we learn that engaging empathically with SPs is not something students navigate on their own in the course of their interactions, but rather something that is regimented. Students are expected to demonstrate empathy in the form of verbal phrases three times to meet the requirements for their assessment.

### 4.2 Implications of engaging with and enacting curricular clinical empathy

Eight students recount instances of empathic dissonance (Laughey et al., 2021) in which they felt “uncomfortable,” “awkward,” “confused,” “frustrated,” annoy[ed],” “hinder[ed],” “weird,” or even “nervous” while practicing empathy. For example, Courtney explains, “They had us say our empathetic statements in a format that just feels uncomfortable to me. So, you had to say, ‘you feel sad, because whatever happened,’ and it just feels a little bit clunky, in my opinion.” Like Courtney, Blake recounts how the empathetic statements “hinder” his ability to engage empathically. He says, “personally, and maybe I’m wrong…I hate being scripted more than anything on the planet, and I think that really hinders me.”

Whereas Blake and Courtney struggle with feeling hindered or uncomfortable, Kyle recalls being frustrated by simulated interaction itself:

My last one (interview) was my worst one. I went in, the patient was acting like he was supposed to have a headache, and he was like, sitting there staring down, like rubbing his temples. He wouldn’t look at me, and he was moaning in pain, and I knew that he wasn't feeling this, so I couldn't empathize with the sounds. Like, “you're just acting.” So, it just becomes more frustrating. And I know they're trying to psych me out. If someone was actually feeling that way, I'd be like, “Oh, my god, that's horrible.”

Kyle explains how not only the empathetic statements, but knowing the SPs are acting, makes it harder to engage empathically—a sentiment that is shared by six of his peers. Kyle and his peers believe if they knew the patient was actually in pain or emotional distress, that they would be able to genuinely feel empathy for the patient and act on those feelings accordingly. In this context, empathy then becomes a form of emotional labor that allows students to meet a curricular goal but at the expense of what they perceive to be their “genuine” feelings.

Referencing their own empathic capital, students in our study contrast the empathy they “fake” with SPs with empathy they perceive to be authentically cultivated from genuine concern and feeling. For Neel and Luke, their conceptualization of empathy is also connected to “care,” and they express how the evocation of that care is not something that is programmable, but rather comes from “being a genuine person” or from feeling the feelings of another (i.e., affective empathy). Neel explains:

I think a lot of the things about empathy are kind of, I don't know, like, at times, it feels so forced to me. It's just like, if a patient says something, you should say “oh, I can't even imagine how painful this is.” I don't know, they just have a lot of these like key words where it's like, this just feels so fake. But I just think a lot of empathy should come from you being a genuine person and truly caring about someone. I guess it (empathy) can be taught to a certain extent, but I think that a lot of people who want to be physicians, I think they have it (empathy) for the most part.

Luke draws on his own definition of empathy as he distinguishes between what empathy *is* and what empathy *is not*. He highlights the importance of the “sharing” and “feeling” that must take place between himself as the empathizer and the patient as an empathy recipient:

You can take the textbook definition of empathy, but for me it's like, at least, sharing in the patient’s experience and feeling how they feel and trying to relate that to your own experience and relaying back to them that you understand and that you care. [It's] not just saying “I'm really sorry to hear that,” like a robot, which is kind of what we're taught.

Luke views empathic engagement as a multidimensional process that involves both cognitive and affective dimensions of empathy. Thus, for Luke, relying on scripted empathetic statements to convey empathy is something a robot could be trained to do and ultimately negates the importance of the interpersonal aspects of empathic engagement.

Two students, Hayden and Kyle, view their empathic abilities as “natural,” something that is at odds with the standardized way that they are asked to express emotion with simulated patients. Hayden explains, “Midtown’s big phrase is ‘you feel because’ so it’s like, ‘you feel (blank), because (blank),’ which is like such an unnatural way for me to be empathic. I would never look at a friend and be like, ‘Oh, you feel sad, because your dog passed away.’” He goes on to say, “I feel like there’s so many ways you can show empathy…I try to use callbacks [recalling information that was shared earlier in the course of an interaction] and I try to show with my body language that I’m listening.” Hayden not only finds the empathetic statements of curricular CE to be unnatural but also uninformed by the empathic capital that he and his peers bring to the table when the matriculate into medical school.

Similarly, Kyle reflects on his own empathic resources:

I always felt like I wanted to practice empathy in my own way; like how I felt I should be empathetic. And then they were kind of pushing their own way…because if you don't really know how to profess empathy, then that's [the curriculum] good. But for me, it just felt like it was kind of conflicting with my natural human instincts.

Kyle points out a central conflict he is experiencing as he feels he is being pressured to adopt Midtown’s approach to empathy over and above his own approach. Also, Kyle tethers his understanding of empathy to his understanding of what it means to be human, noting that this is something he thought he already knew how to do proficiently, that is, until he started his interview skills seminar.

Six M1s contrast instances of momentary empathic dissonance with implications that are, consequentially, influencing their perceptions of how empathy is used personally and professionally, their own notions of the types of providers they want to be, and their views regarding the role of emotions in medicine. For example, prior to his interview skills course, Blake was under the impression that having empathy in the context of interactions with others is the same whether you are inside or outside of a clinic. He recalls an instance in which he had to reevaluate that perception. He says:

I remember talking to my friend and I was like, “Well, I'm really sorry about how that happened.” And I like kind of, you know, took a second look at it (and thought) Why the hell did I just use that with a good friend?…It wasn't even something that was serious. You know, they didn't have what you wanted on DoorDash…I didn't need to use a doctor voice for that. I understand. It sucks. You didn't get your breakfast sandwich. So yeah, I do think that I'm going to have to find a way to separate the way that I communicate with patients and my life outside of medicine.

Blake is obviously shocked by the unconscious integration of curricular CE methods into a casual conversation with a friend. What he deems the “doctor voice” is now a programmed response that he found unnecessary, because he conveyed concern for something that he perceived to be inconsequential. As a result, Blake deduces that he needs to be mindful of and even distinguish between the way he communicates empathy in his personal and professional lives.

For Aron, simulated patient training has actually diminished the degree to which he believes emotions and empathy matter in medical care. Aron says:

I have a lower opinion of emotions in the practice of medicine than I did at the beginning of the study just because, you know, the fake interviews. We’re practicing faking emotion, like faking empathy, so now it doesn't really matter, one way or another to give them empathy, because, you know, we’ll still treat them for whatever.

Similarly, Courtney questions whether her predilection to “be there for patients” is of consequence in her training and future practice:

I guess I had kind of a naive view that maybe it would be accepted that I would want to be totally myself and like, be there for patients, and I think, at least so far, what I've experienced and seen and talked to other people that maybe it’s (her implementation of empathy) not as welcomed as I thought it would be.

For Abbey, the problem is that medical school requires students to perform empathy even when they cannot relate or understand a patient, something she finds perplexing:

You know, empathy is trying to relate to the patient and put yourselves in their shoes, and trying to make them feel like you understand, but sometimes you don't understand. You just can't fake that…I feel like they push faking empathy a lot when they should just let you, I don’t know…It’s just rules I don’t understand.

Abbey is making a crucial point with respect to clinical empathy: There are times when a provider cannot understand a patient’s perspective, because of power imbalances, bias, or simply miscommunication. In these moments, *knowing that you do not know* may be as much evidence of emotional capital as “faking” a connection or understanding. This reality can be obscured when the emphasis is performance rather than a student’s capacity to be reflexive about a given situation.

What is clear from the accounts presented above is that students’ empathic capital often bumps up against the standardized empathy training offered at Midtown. This leads students to feel empathic dissonance either because they perceive their genuine capacities for empathy are underutilized or because they are being asked to perform empathy when in fact they do not feel it. Common across these trainee accounts is a belief that feeling *genuine* empathy for patients is the gold standard of care. They take umbrage at the idea that empathy might—at times—be “faked” by providers.

While addressing implications of curricular CE, it is important to note that six students did not find fault with CE in the context of interview skills training. Even though the majority of students criticized the curriculum, a total of 10 students, including students who were critical, admitted that the things they learned in the context of interview skills training have become more natural and have been useful in (1) helping them or their peers listen intently to others, and (2) helping them to better express the empathy they are genuinely feeling. Students even note how they intentionally use empathetic statements in interactions with family and friends, and how those empathetic statements can be useful in the context of a real patient interaction.

### 4.3 Medical educators’ perceptions of student empathic capital

Two-thirds of the MEs in the study (*N* = 6) spoke of the empathic capital or emotional intelligence that students bring to the table; all agree (*N* = 9) that these skills and resources are not uniformly distributed in the student population. For example, Dr. Olsen says, “having worked with students in the interviewing course, you can see students, they have it [empathy] very well there, and some who really struggle with that awareness [of others].” She goes on to discuss her experience teaching the interview skills course and explains why some students struggle:

To come in and work with the students is very like (she laughs), “Oh, my god; we have a long way to go.” You know, they are looking so concretely [at] things…More often than not, a patient's like, “I'm coming in for this”—you know, they have a coached patient—“I'm coming in; I want to stop smoking.” I think it's one of their constant cases (she laughs), and they (students) would be like, “That must make you frustrated.” [SP]: “I can't quit smoking.” [Students]: “That must make you frustrated,” and they use the same word over, and over again. So, they're really not thinking about all the other pieces to the person's presenting issues. Like, their teenage daughter's going off the rails, and their work is difficult. So, there's stressors that relate to a coping mechanism that increases the smoking habit…but it's hard for them to draw the connection.

Dr. Olsen describes the M1s in her class as “concrete” thinkers and notes how that inhibits their ability to think broadly about the psychosocial experiences of future patients and how those experiences contribute to various health behaviors and outcomes.

Implicit in MEs perceptions of students’ empathic capital is the belief that empathy is acquired through the depth of lived experience, something MEs believe students generally do not possess. Dr. Upshaw explains:

I think the more pain you go through, the better you are at giving empathy. When you've been through enough experiences—some people would argue that the reason you go through those things is so that you might be of help to somebody who's going through something similar. I think, not that young people can't give empathy, but people who've been through more in life, they seem more understanding…And I'm not being ageist, but it just comes with experience, I think…Of course, you can be empathic as a young person and say, “Oh, that's horrible,” and then when you actually live through them (various experiences), you say, “Oh, that was much worse than I even imagined.”

Not unlike Dr. Upshaw, Mr. Parker is under the impression that for those M1s who are not as empathically inclined as some of their peers, a lack of work experience is to blame. He says:

I guess my expectation of medical students is that they were like fully formed adults; they had their shit together (he laughs)…and there’s a lot of difference. Some of them have had a lot of life experience, and some of them are comfortable, others are not. So, I see that those that haven't had work experience…or those students that did two years of undergrad, a lot of them have never had jobs…Like, I learned so much of empathy from waiting tables, from just interacting with people, getting those people skills, and if they don't have that type of foundational experience of a lot of interaction with humans, and other than just like with their family, I think it's hard to understand what empathy is at that first year of medical school and to truly be able to do it. I think it happens eventually, but certainly students are at different points.

Though the MEs in this study do not consider M1s to be blank slates when it comes to empathic ability, they perceive that some students have more empathic capital and lived experience than others, and thus, consider the implementation and implications of their curriculum accordingly.

### 4.4 Medical educators’ perceptions of curriculum

Medical educators are not oblivious to students’ perceptions of the empathy training curriculum as rote or “fake.” Four MEs reference their awareness of students’ critiques and even echo students’ impressions of the curriculum, especially when referencing the scripted empathetic statements. Ms. Nielsen, for example, explains, “the way we teach it (empathic interviewing), it seems robotic. I know sometimes that’s a challenge to try to get people (students) to get over that hurdle of being robotic.” Though MEs echo students’ critiques, they are quick to defend the curriculum. According to Dr. Abbott, the process of “filling in the statement ‘you feel because’ to get at empathy” is “really corny and hokey,” but he maintains that that “is the essence of what an empathic response is all about.” Dr. Abbott is underscoring the importance of not only conveying empathy effectively, but accurately.

Drawing on his own clinical experience with Midtown’s empathetic statements, Dr. Upshaw attests to the effectiveness of Midtown’s approach. He explains:

Empathy can become formulaic, especially in the way that we teach and test…The formulaic approach is…actually very effective. Say, after the patient has told the story, you can say, “Oh, you feel angry, because this person betrayed you,” and it's sort of a “yes, you nailed it! Yes, I do feel angry.” So, that's probably one of the better formulaic approaches.

Medical educators further justify overcoming the “hurdle” of practicing robotic empathy by explaining it to their students, in essence, as *a means to an end*. Like other affective practice approaches discussed in the literature, MEs at Midtown purport that the more time students spend practicing with these statements, the more natural (unconscious) or genuine their empathic engagement with patients will become. In short, MEs see value in “faking it until you make it” and, unlike the students, are less concerned that trainees tap into genuine or felt empathy, at least initially. MEs reassure students that CE will get easier with practice. Ms. Nielsen tells her students, “It’s like learning to crawl before you walk. I tell them, ‘we are going to have you use these specific tools to kind of get that feel’…I really tried to preface it as, you have to do this before you can get here.”

Dr. Hayes and Dr. Abbott liken curricular CE training to the experience of learning to ride a bike. For example, Dr. Hayes explains, “they [empathy statements] can be great training wheels, because they remind you that you are supposed to say it and, at some point, it becomes more genuine when you yourself become more comfortable in your practice.” Similarly, Dr. Abbott believes that students get to a place of genuine, almost instinctual, concern for patients only after they have moved through the necessarily awkward stages of consciously practicing empathy. He says:

I would say, if I'm doing it [empathy] more consciously, it's less genuine, it's less effective, it's less really authentic [than] when it just comes naturally. And that's what we want to get the students to a point. It’s kind of like riding a bike. You start with training wheels, and it just feels awkward and you're trying to keep the thing up. Then you take the wheels off; you're trying to keep the bike balanced…And to be truly empathic is, you don't think about it anymore. You are being empathic…And so, in real, authentic conversation, you can do this with your friends, with confidants. If you're a counselor, that (being empathic) happens in a real context with a client.

Medical educators recognize that students have empathic capital prior to coming to medical school but educators perceive this capital as inadequate for the purposes of doctor-patient communication. Ms. Nielsen explains:

A lot of times students come in, and what I've seen throughout the years, is that they come in, and they're like, “I know how to talk to people, so why do I have to do this?” And then when they actually go into the rooms (in the simulation lab), they're like, “this is different.” And it's like, “it *is*, it's a professional way of talking to somebody; it's *not* they're your buddy, they're your friend, you know?”

For some MEs, the fact that students have empathic capital can actually blind trainees to the perspectives of patients, which is anathema to the “patient first” mentality espoused by educators. As Dr. Henry puts it:

The patient comes first. Even though you have that set of values, you have your own personal experiences and backgrounds and belief systems, and even if you don't agree with that patient, you're always putting them and their needs first. I try to guide students saying, “hey, you know, you got to get to a place where you're understanding the patient.”

Similarly, Dr. Abbott cautions that when students approach patients with the intention to relate emotionally, based on their own lived experience, it can undermine the goal to really listen and understand patients:

The first thing that they (M1s) do is learn to just sit in and sit with a patient and really listen to them and hear their story. It's a hard thing to do…It's very difficult to take the time and turn yourself off, you know, mostly, all of us. It's human nature to want to tell your story. But what most people do is go right to, “Well, yeah, you know what, the same thing happened to me. Oh, your uncle had a heart attack. Well, geez, that happened to my dad some years ago, and here's what I did.” And suddenly, who's it about? It's not about you anymore, it’s about me and my story, and what we want the students to do is to shut themselves off, and give themselves over to the patient, and really listen and hear.

Medical educators also believe that centering the patient in this way allows providers to keep patients satisfied and coming back for care. The goal, then, is not necessarily to feel genuine empathy as a provider but to construct a positive experience for patients so that they do not “vote with their feet” and go elsewhere for care. Ms. Nielson explains:

So, to really be a good health professional, you got to talk to people and understand who they are…I don't think you can create that relationship fully without empathy…With your health profession [al], you want to be able to relax with them, you want to feel comfortable, you want to be able to say, “I can share anything with you,” and so you have to have that relationship to do that…I put it in this sense too, for the med students, I’m like, “do you know how many doctors there are out there? If somebody doesn't like you, or doesn't feel like they connect with you, they can always go somewhere else.” I mean, it kind of is almost like that business sense as well. If I want to keep my clientele, I have to know how to interact with them. So, I think that's helpful. And that's why I think it's (empathy) a big thing too.

Medical educators are acutely aware that there are times when it becomes challenging to inject empathy into a clinical encounter. Trainees must learn how to engage empathically with patients even when a genuine connection is impossible or difficult to achieve. Dr. Henry, for example, draws on her understanding of concepts she has coined “(*little*) *e”* and “(*big*) *E”* empathy. Both are empathy, but *(big) E* involves addressing or treating all aspects of the patient—physical, social, spiritual—whereas *(little) e* still involves connecting with the patient, but perhaps, out of necessity, only concentrating on one dimension of their care. Both *(big) E* and *(little) e* can be influenced by length of relationship as well as other circumstantial variables, and both reflect the concepts of surface and deep acting *a la* Hochschild (1983). With this understanding, Dr. Henry outlines how CE is instrumental in those moments. She recalls telling her students:

Look, there's going to be times where you're not going to connect with your patients at all, and that's okay. There may be a patient that is belligerent. They're experiencing distress, they're afraid, they're embarrassed, you know, and they are lashing out. They are calling you out based on, you know, skin color, or gender, or whatever it is, and you can't connect with that patient, because you've been hurt, you've been harmed, there's been something in that even developing relationship where you're shutting down, and you're like, “Okay, I'll go through the motions, because I have to, rather than I want to.” Then I sort of say, okay…then that's the (*little*) *e*; you're gonna do your best…When you have a patient that is offensive, or belligerent, or something, it's not caring about that patient, it’s caring about you to protect yourself, and it's caring about, “I have to fulfill a duty because I'm obligated to.”

In summary, MEs understand medical student desires to feel and enact genuine empathy in the clinical encounter, and they are aware of trainee frustration with the rote nature of CE curriculum. Educators, however, believe that there is value in “faking it until you make it,” not only for the provider but for the patient as well. Standardized and performative empathy has its place in medicine, according to educators, for it ensures that the focus remains on patient experience (rather than the provider’s), increases the likelihood that patients will return for care, and also gives providers tools to engage in “(*little*) *e*” empathy when they do not have the bandwidth or inclination to connect genuinely with patients.

### 4.5 Medical school as an unkind place

Medical educators and students are in complete agreement when it comes to one thing: The toll medical professional training model exacts from students, specifically regarding the demands of the biomedical curriculum. M1s in our study describe various facets of the biomedical curriculum at Midtown as “crazy,” “frustrating,” and “ridiculously hard.” For example, Briti recalls the experience of anatomy class. She explains, “I think over half the class failed the (anatomy) exam…It was just ridiculously hard. We had anatomy in 4 weeks…so you really are working your butt off those 4 weeks…You can probably talk to anyone; it was a really rough time.” Dr. Abbott echoes the students’ impressions of the curriculum, saying “the students are crushed by the curriculum, because it’s so demanding. You can just see it in how they carry themselves…It’s just taxing; the science courses are really tough.”

Medical educators and M1s discuss the discomfort and frustration students experience as they try to ascertain where to focus their time and energies with regard to the demands of medical school curriculum. Dr. Olsen explains how students navigate this process:

Anything that is not core to them, you know, having time and space dedicated to their studying to keep up with the amount of curriculum that they need to, they feel is secondary, and so they're very frustrated as a whole, because they feel like they don't have enough time designated to be able to (keep up). The amount of information that they have to consume and memorize is so hard in the way it's structured…They get frustrated that anything that is considered cupcake and not relevant…If it's not high yield, and it's not anything directly relating to their education and getting them a better score on their medical exams, or getting into the residency of choice, it is not priority.

Medical students are forced to prioritize their science classes over and above their own wellness and the human values and interview classes they find valuable. For example, Dr. Thomas says:

The students may not be in that frame of mind to think about how broader social factors influence health. They're really focused, by design of their curriculum, on cells, biology, anatomy, chemistry, all of that stuff to get to the physiology of how the body works and functions and dysfunctions.

Dr. Olsen addresses M1s relationship with wellness and the curriculum, noting:

What wellness was like to them (M1s) before they started medical school is vastly different than where it is now. You know, you might be able to fit in an hour a day, you know, workout, you might be able to see friends and hang out, see your family. When you go into medical school, you feel like all those doors kind of close up.

Medical students Ginni, Briti, and Blake reflect on their experiences attempting to balance the requirements of their sciences courses with the requirements of the humanities and interview courses. Ginni explains:

I like human values, but I also really like reading stories…I enjoy writing, I think too…I enjoy reflecting and things like that. If you ask my friends, they'd be like, ‘this is useless. We have so much science things to do. Why do I have to write an essay? Which, sometimes the timing was kind of awful—right before a huge exam, and we'd be like “I don't want to be reading and writing when I could be, you know, looking at cadavers.”

Blake recalls a week in which they were responsible for three cumulative final exams, all worth more than 25% of their final grades, a “hell of a schedule,” he says. He goes on to say:

After that first exam, they made us attend a mandatory two-hour long poetry session that was just, I was just so mad about it. And that's, you know, one of the cruxes. I love the humanities; I think all these things are so important, and one thing that really makes me so mad is I don't get any time to actually delve into them and experience them, and just be there in the moment, because there's always something else going on.

Like Ginni and Blake, Briti recounts feeling like she could not put any effort or focus into her humanities and interview seminars. She says:

Sometimes we would have those sessions (humanities and interviewing skills) right before an exam…or we would have an interview the day of our exam. It became more like a checkbox thing, like, “Okay, did you write something on a piece of paper for your essay? Okay, turn it in. Okay, go to the group discussion.” I feel like that took the meaning out of it because we're so tired from studying.

With science courses taking precedence, MEs and M1s are cognizant of what this means for student empathy development. For example, Dr. Abbott explains:

The system we (administrators and faculty in medical education broadly) set up does not promote their (students’) wellness, it doesn't promote their focus on being more empathic because they feel distressed. So, I think even though we're teaching this (CE) and supporting them as much as we can…the [biomedical] curriculum has an effect in the first year.

Dr. Abbott recognizes that even with the educational interventions designed to enhance empathy in the context of interview skills training at Midtown, the impact of the broader curriculum forestalls the development of students’ wellness and empathy. Similarly, Dr. Garrett discusses the impact of the curriculum and his attempts to support and even empower students, encouraging them to fight for their own caring and empathic inclinations. He says:

My own theory…is that learners come to this enterprise with a great deal of empathy and compassion, but the educational process is deleterious to their own level of empathy and compassion…I tell students all the time: (he asks them) “Why are you here? Because you care about suffering people? Okay. We're going to do everything we can to beat that out of you in the next four to 10 years, and your job is to try to keep us from doing it. So, you got to figure out ways to hold on to how you want to be in medicine as an empathic, caring, loving person…if that's how you want to be, how are you going to hold on to it?” A lot of our educational efforts here are aimed at giving them space to try to build that reflective capacity.

It is important to note that no M1s in our study ever questioned the value of relational skills courses like CE and humanities, but students were skeptical about their usefulness with regard to time, especially when compared to the amount of material they were responsible for in their science courses. Jabarr notes, “they are trying to teach us to be humans and be normal, you know? But I feel like I can interview patients fine. It just seems like an excessive use of time. I had rather be doing something else, but I do see the value in it…” As the M1s explain, given the choice between concentrating on their science courses and participating in their relational skills courses, science courses take precedence. Further, because students are forced to prioritize the accumulation of scientific knowledge over their own wellness and their own perceptions of what is actually valuable in the curriculum, they are subject to a hidden curriculum that positions success in biomedical coursework above all else, including above their own physical and mental well-being.

## 5 Discussion

Consistent with existing scholarship, we find that medical trainees at Midtown view the standardized training in CE to be robotic and forced. This experience fosters a sense of empathic dissonance (Laughey et al., 2020a), wherein students are asked to engage in insincere expressions of empathy (the “doctor voice”) that either contradict their internal emotional state or do not validate or draw upon their own empathic capital. Some students, like Courtney, express concern that this kind of performative empathy (what Underman calls, “affective practice”) negates her own ways of interacting with others in the world, but also subordinates her true sense of self while on the job. Similar to the flight attendants in Hochschild’s classic work, *The Managed Heart*, one potential consequence of this emotion management is the sublimation of one’s more “authentic” self, with potential implications for trainee job satisfaction, burnout, and mental well-being down the road (Hochschild, 1983).

Medical educators working at Midtown are not blind to the emotional capital that trainees bring to the table, and they strive to create a curriculum that works for all students, given that students’ primary emotional socialization is highly variable. Educators view empathy as something one gains with maturity and tend to see medical trainees as lacking in meaningful emotional experiences prior to medical school. Of course, educators are not misguided in the view that a 20-year-old student will have less emotional capital than, say, a 40-year-old primary care doctor. However, it is worth noting that student trainees *perceive* that they have meaningful experiences (or emotional capital) that shape their ability to engage in CE and they bring these perceptions with them as they “practice” empathy with simulated patients. As such, there is a discrepancy between how the educators and students perceive the empathic resources on the table during CE training.

Educators recognize that CE training is at times “robotic,” but they believe that over time students become clinicians who eventually possess and enact more genuine and “natural” expressions of empathy. In short, they see value in “faking it until you make it” when it comes to empathy. At the same time, educators view CE as a patient centered affair, which may at times require students/providers to “shut themselves off” and perform empathy such that they can achieve clinical and interactional goals in the context of an encounter with a patient. Knowing when to employ “(*big*) *E*” empathy versus “(*little*) *e*” empathy is crucial to achieving quality patient care, especially in those moments when genuine concern for a patient cannot be summoned. Similar to the nurses discussed in the book *Practical Feelings* Cottingham (2022), medical educators view empathy as an emotional resource that, at times, must be conserved and shared judiciously. Although not explicitly instructing students to conceptualize empathy as emotional labor, MEs understand that there are times when a “surface acting” (Hochschild, 1983) approach to CE is sufficient and sometimes even necessary to successfully manage the demands of a career in medicine.

Perhaps most notably, both students and medical educators recognize that empathy training often takes a back seat to the demands of biomedical training. The pre-clinical and clinical years of medical school require students to sacrifice self-care, personal relationships, and extracurricular interests, all in the name of successfully matriculating through crucial phases of medical school (e.g., STEP exams, clinical rotations and shelf exams, and residency placement). The biomedical curriculum takes priority over the relational skills training of empathy development and medical humanities. While students and instructors alike understand (and even relish) the opportunity to read poetry or contemplate patient experience, the structural constraints of a biomedical curriculum means that they must make decisions about where to place finite intellectual and emotional energies. Not surprisingly, CE takes a back seat. In short, medical students are asked to ‘be kind in an unkind place,’ a reality that will follow them into their careers as physicians working for health care organizations where the requirement to be compassionate exists alongside high patient loads, checks on provider autonomy, and speed up of work. Our findings bear eerie resemblance to the observations of Becker et al. (1961) who, over 60 years ago, described the ways that students reconciled their idealistic professional goals (i.e., helping people) with the realities of pre-clinical training by making strategic decisions about what constitutes “important” information (Becker et al., 1961, p. 111; Becker and Geer, 1958).

This last observation is particularly important as we imagine ways to foster CE in medical students moving forward. Borrowing from conceptualization of emotions as practice of Cottingham (2022), we suggest that medical schools might benefit from explicit recognition (among faculty and with students) of the ways that: (1) empathy is a resource or form of emotional capital that students already possess as a result of primary socialization and that students perceive this capital to be valuable to their training, (2) emotions and the management of those emotions is integral to—rather than adjacent to—becoming a doctor; and (3) there are institutional and organizational norms and constraints in medical school that powerfully limit how trainees learn to *feel* and enact *feeling* in medical school.

Although instructors at Midtown clearly recognize the empathic capital that students bring to medical school from primary socialization, they tend to minimize that knowledge in favor of standardized training, believing that genuine empathy will come in time and that students should focus on standardizing their approach to communication. Recognizing that “emotions are practical, embodied calculations haunted by past practices and predictive of future demands” (Cottingham, 2022, p.162), we posit that first and second year empathy training should begin by explicitly recognizing the empathic capital that students bring to the table and integrating those understandings into empathy training. In so doing, the curriculum is simultaneously student generated and “owned” but also attuned to the reality that some students more than others need pat language and rote learning to truly understand how to empathically engage with patients. A first step is for instructors to solicit student understandings of empathy at the outset of M1 year and work explicitly and in collaboration with students to validate and develop this emotional capital over time. Doing so would move clinical empathy training closer to what Rebecca Olson and colleagues have dubbed, “emotionally reflexive labor” (Olson et al., 2021, p. 17), which acknowledges student “capacity to interpret their own and others’ thoughts and emotions.”

Of course, changes to specific empathy/interviewing courses does little to address the structural realities of medical school, which prioritize biomedical learning over social–emotional skills development. Students will never invest seriously in empathy development in a context where the dualisms of reason/emotion and thinking/feeling profoundly shape the learning environment. Moreover, it is difficult for students to develop their own sense of empathy and compassion when their own professional development takes place in an environment of ritual hazing and shame, with intense pressures to excel and compete. Midtown certainly works hard to provide buffers of support for students in this context, but at the end of the day the school is held accountable to the number of students who successfully pass STEP exams and find a residency spot [both of which are determined by boards and organizations that set standards for medical education, such as the Association of American Medical Colleges (AAMC)]. Until those larger bodies integrate emotional intelligence and socialization into curriculum and testing, medical schools will continue to offer relational skills curriculum, knowing full well it takes a back seat to biomedical courses. As such, medical schools—and the students within them—will feel as though they are swimming upstream in an attempt to cultivate empathy and kindness in trainees.

The current study is not without its limitations. The sample of students interviewed is small and any findings discussed here likely reflect the idiosyncrasies of the particular learning environment of Midtown. Another medical school with a different approach to empathy training may yield different student/faculty perceptions. Additionally, we rely here on student accounts of their empathic capital, rather than observational or survey data that might measure their empathy in more objective ways. Future research should consider triangulating student and faculty perceptions of empathy training with ethnographic observations of classroom instruction and simulated patient experience. Finally, this paper focuses on empathy training at one point in the medical career of students. Longitudinal data is needed to fully understand how empathic capital accrues—or is depleted—over the course of 4 years of medical school.

Despite these limitations, our findings suggest that medical students possess considerable empathic capital and that their instructors go to considerable lengths to foster learning in the area of social–emotional intelligence. The larger context of medical education, with its focus on matriculation assessments and residency placement, leaves little room for students and instructors alike to generate and participate in curriculum that develops clinical empathy in a sustained and meaningful way.

## Data availability statement

The datasets presented in this article are not readily available because the data for this paper are qualitative. In accordance with human subjects regulations data for this study has been de-identified and is confidential, but cannot be fully anonymized. Thus, in order to protect the confidentiality and privacy of research participants, data for this study are not readily available. Requests to access the datasets should be directed to SH, sharve10@kent.edu.

## Ethics statement

The studies involving humans were approved by the Institutional Review Board at Kent State University. The studies were conducted in accordance with the local legislation and institutional requirements. The participants provided their written informed consent to participate in the study and for the publication of any potentially/indirectly identifying data included in the article.

## Author contributions

SH: Conceptualization, Formal analysis, Funding acquisition, Investigation, Methodology, Project administration, Writing – original draft, Writing – review & editing. CS: Conceptualization, Formal analysis, Funding acquisition, Investigation, Methodology, Project administration, Supervision, Writing – original draft, Writing – review & editing.
